# Comparative Study of Clinical Characteristics According to Pain Distribution in Lingual Burning Mouth Syndrome

**DOI:** 10.1111/joor.70269

**Published:** 2026-07-15

**Authors:** Yoohyun Sim, Na‐Yeon Ha, Seok‐Jae Ko, Jae‐Woo Park, Jinsung Kim

**Affiliations:** ^1^ Division of Digestive Diseases, Department of Korean Internal Medicine Kyung Hee University Korean Medicine Hospital Seoul Republic of Korea; ^2^ Department of Clinical Korean Medicine, Graduate School Kyung Hee University Seoul Republic of Korea; ^3^ Department of Digestive Diseases, College of Korean Medicine Kyung Hee University Seoul Republic of Korea; ^4^ Department of Korean Internal Medicine Kyung Hee University Hospital at Gangdong Seoul Republic of Korea

**Keywords:** lingual burning mouth syndrome, pain, quality of life, salivation, taste disturbance

## Abstract

**Background:**

The pathophysiology of burning mouth syndrome (BMS) remains unclear.

**Objectives:**

To compare the clinical characteristics of patients with lingual BMS according to pain distribution.

**Methods:**

The medical records of patients with lingual BMS who visited the oral clinic of Kyung Hee University Korean Medicine Hospital between January 2023 and December 2024 were retrospectively reviewed. Patients were classified according to tongue pain distribution as follows: laterality (unilateral vs. bilateral), extent (localised vs. generalised) and central tongue involvement (non‐central vs. central involvement). Group differences and associations among clinical characteristics, including pain intensity (visual analog scale [VAS]), quality of life, salivary flow and taste disturbance, were assessed.

**Results:**

In total, 256 patients were included. Patients with bilateral pain showed significantly higher pain intensity than those with unilateral pain. Patients with generalised pain reported significantly higher pain intensity than those with localised pain. Patients with central involvement showed significantly higher pain intensity than those with non‐central involvement. Pain intensity was negatively associated with quality of life in most subgroups, except the unilateral subgroup. Taste disturbance was associated with clinically significant pain (VAS ≥ 4) in the unilateral and localised subgroups, whereas reduced salivary flow was associated with clinically significant pain in the central involvement subgroup.

**Conclusions:**

Pain distribution may help identify clinical subtypes of lingual BMS and support individualised assessment. Herein, pain distribution was associated with differences in pain intensity, quality of life, taste disturbance and salivary flow, underscoring the heterogeneous and multifactorial pathophysiology of lingual BMS and the need for tailored management.

## Graphical abstract

Tongue pain mapping in 256 patients with lingual burning mouth syndrome identified distinct clinical phenotypes according to pain distribution. Wider pain areas were associated with higher pain intensity and poorer oral health‐related quality of life, supporting individualised assessment and targeted management.

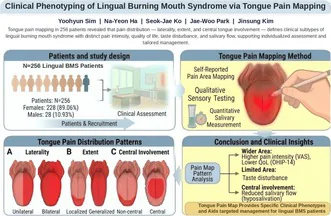

## 1 Introduction

Burning mouth syndrome (BMS) is a chronic oral pain condition characterised by persistent burning, stinging or dysesthetic sensations in the oral cavity, despite the absence of clinically evident oral mucosal lesions. According to the International Classification of Headache Disorders, 3rd edition (ICHD‐3) proposed by the International Headache Society, BMS is defined as an intraoral burning or dysesthetic sensation occurring for more than 2 h per day and persisting for more than 3 months [1, 2]. The prevalence of BMS has been reported to range from 0.7% to 15% in the general population, and it occurs 2.5–7 times more frequently in women than in men, particularly among peri‐ and postmenopausal women [2].

The pathophysiology of BMS has not been clearly elucidated; however, peripheral nerve injury, small fibre neuropathy, sensory nerve dysfunction, central sensitisation, autonomic imbalances, hormonal changes and psychological factors have been suggested to be involved in a multifactorial manner [2]. This pathophysiological heterogeneity suggests that BMS may represent a heterogeneous syndrome encompassing different mechanisms and clinical presentations rather than a single disease entity.

Clinically, pain is localised to the tongue in approximately 68% of patients with BMS, most commonly affecting the anterior two‐thirds of the tongue and typically presenting as bilateral burning pain [2]. However, unilateral symptoms have been reported in approximately 20% of patients [3, 4]. Although the distribution and laterality of pain provide important clues for inferring the pathophysiological mechanisms in neuropathic pain conditions, studies that systematically classify patients with BMS according to their pain distribution and compare their clinical characteristics are scarce. One prior study, conducted in 2020, compared patients with BMS according to pain distribution (bilateral vs. unilateral) and found that patients with bilateral pain more frequently exhibited hyposalivation (78.3% vs. 67.0%, *p* < 0.05), taste disturbances (51.7% vs. 37.1%, *p* < 0.05) and psychological distress, whereas patients with unilateral pain more frequently reported dental treatment as a precipitating factor (21.4% vs. 47.4%, *p* < 0.001) [4]. However, that study was largely limited to questionnaire‐based assessments and did not examine the associations among clinical variables.

The present retrospective study sought to address these limitations by reviewing the medical records of 256 patients with lingual BMS and classifying the patients into subgroups according to their pain distribution patterns. The patterns were based on three criteria, that is, pain laterality, extent of pain and presence of central tongue involvement. These subgroup criteria were selected to reflect different spatial characteristics of tongue pain and to facilitate exploratory comparisons of clinical features according to pain distribution patterns in lingual BMS. Associated clinical factors were compared across the subgroups. Through this approach, we aimed to explore the potential for classifying clinical subtypes of lingual BMS based on pain distribution patterns and provide a foundation for more precise diagnosis and individualised treatment strategies.

## 2 Materials and Methods

### 2.1 Study Design and Ethics

This retrospective study included patients with lingual BMS who visited the Oral Disease Clinic of the Department of Gastroenterology at Kyung Hee University Korean Medicine Hospital, Republic of Korea, between January 1, 2023 and December 31, 2024.

The study was conducted in accordance with the Declaration of Helsinki and approved by the Institutional Review Board of Kyung Hee University Korean Medicine Hospital (IRB No. KOMCIRB 2025‐08‐016, approved on August 22, 2025). The requirement for written informed consent was waived by the Institutional Review Board due to the retrospective nature of the study. This study involved the review and analysis of existing medical records, including questionnaire data and examination results, of patients who had completed clinical assessments within the study period. No direct contact with participants was conducted.

### 2.2 Inclusion and Exclusion Criteria

Participants were adults aged ≥ 19 years who visited Kyung Hee University Korean Medicine Hospital between January 1, 2023 and December 31, 2024, and were included if they met the diagnostic criteria for BMS according to the ICHD‐3 proposed by the International Headache Society [1] and had burning pain involving the tongue. BMS was defined as intraoral burning or pain occurring for more than 2 h per day for over 3 months without an identifiable cause, in the absence of visible oral mucosal lesions and without clinically detectable abnormalities on routine oral and sensory examinations. The diagnostic framework was based on ICHD‐3 criteria, which substantially overlap with the International Classification of Orofacial Pain, 1st edition (ICOP‐1) definition of primary BMS. Clinical evaluations, including comprehensive oral examinations, routine clinical sensory testing of the oral mucosa and tongue using light mechanical stimulation with a tongue depressor, meticulous medical history taking and laboratory testing when clinically indicated, were systematically performed to identify and exclude potential secondary local or systemic causes of oral burning symptoms (such as oral candidiasis, nutritional deficiencies and gastroesophageal reflux disease). Patients with clinically identifiable traumatic neuropathy, obvious neuropathic conditions or clinically significant dental or periodontal diseases associated with oral pain were excluded based on clinical history and examination findings. In addition, only patients who indicated their pain location diagrammatically on the BMS questionnaire completed at the initial visit, allowing analysis of pain distribution, were included in the study.

Patients were excluded from the study if they met any of the following criteria: presence of severe systemic diseases, including malignancy or significant cardiac, hepatic or renal disorders; identifiable local or systemic causes of oral burning symptoms, including inflammatory oral mucosal diseases (e.g., oral lichen planus or oral ulceration), clinically significant dental or periodontal diseases, hematologic abnormalities or other secondary causes identified during clinical evaluation; or incomplete baseline assessment at the initial visit. Patients who did not undergo any of the following evaluations were also excluded from the study: pain intensity assessment using the visual analog scale (VAS); evaluation of salivary function; assessment of taste disturbance, including taste hypersensitivity or dysgeusia; and quality of life assessment using the Oral Health Impact Profile‐14 (OHIP‐14).

### 2.3 Data Collection From Electronic Medical Records

Data were collected from electronic medical records based on the clinical documentation obtained at the patient's initial visit. The demographic characteristics included age (years), sex and disease duration (months). Clinical characteristics and symptoms related to lingual BMS were assessed using the visual analog scale (VAS) for oral burning sensation and pain, pain location, presence of taste disturbance and presence of globus sensation (a persistent sensation of a lump or foreign body in the throat). Furthermore, the unstimulated salivary flow rate (USFR; mL/min), which reflects salivary secretory function, was used as an objective clinical parameter [5]. Quality of life was assessed using the OHIP‐14 [6]. The participants were classified into subgroups according to their pain distribution patterns, which were based on the following three criteria: pain laterality (unilateral vs. bilateral), extent of pain (localised vs. generalised) and presence of central tongue involvement (non‐central involvement vs. central involvement) (Figure 1). This classification system was designed to evaluate broader tongue pain distribution characteristics based on standardised tongue pain mapping collected at the initial visit. More detailed anatomical subclassifications, such as tip‐only or anterior tongue involvement [7], were not separately analysed because further stratification would likely have resulted in substantially smaller subgroup sizes and reduced statistical robustness. For patients reporting pain in midline tongue regions (e.g., central, tip or root of the tongue), laterality could not be clearly determined; therefore, these patients were excluded from the laterality subgroup analysis.

**FIGURE 1:**
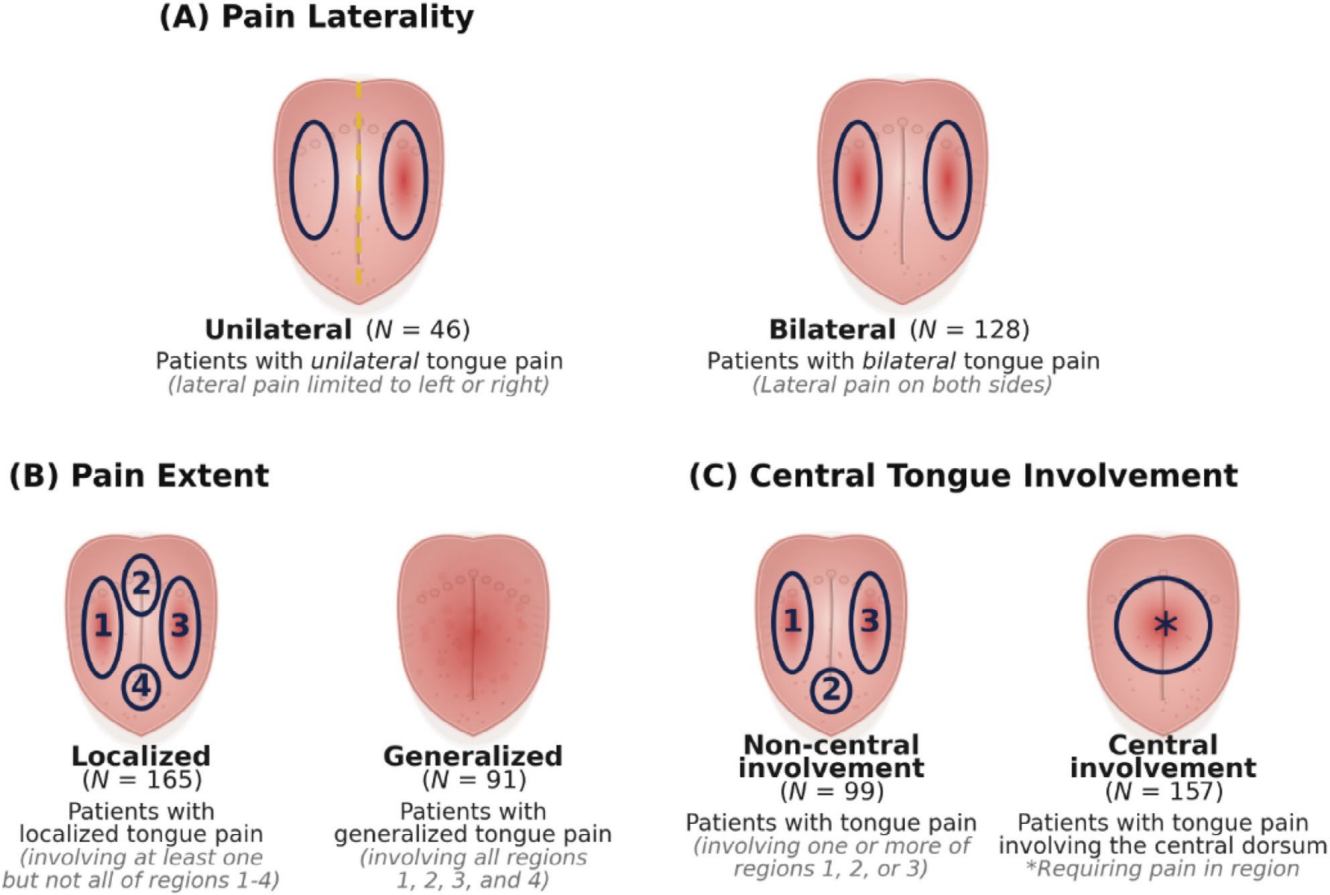
Classification of tongue pain based on laterality, extent and central involvement. (A) Pain laterality was categorised into unilateral (limited to the left or right side) and bilateral. (B) Pain extent was divided into localised (involving at least one but not all of regions 1–4) and generalised (involving all regions). (C) Central tongue involvement was distinguished between non‐central (limited to regions 1, 2 or 3) and central (requiring pain in the central dorsum, marked with an asterisk).

### 2.4 Statistical Analysis

All statistical analyses were performed using IBM SPSS Statistics version 25 (IBM Corp., Armonk, NY, USA). Continuous variables are presented as mean ± standard deviation, and categorical variables are presented as number (%). Statistical significance was set at *p* < 0.05. Differences in clinical variables between the groups were analysed using the chi‐square test for categorical variables and the independent *t*‐test for continuous variables. Given the relatively large sample size, parametric analyses were considered appropriate based on the robustness of independent *t*‐tests to moderate deviations from normality under the central limit theorem. Simple linear regression analyses were performed to evaluate the associations between pain intensity (VAS) and other clinical variables, including taste disturbance, globus sensation and reduced USFR (hyposalivation). Moreover, binary logistic regression analysis was conducted to identify factors associated with clinically significant pain (VAS ≥ 4 on a 10‐point scale). Clinically significant pain was defined as a VAS score ≥ 4, corresponding to at least moderate pain intensity based on previous studies on chronic orofacial pain [8].

## 3 Results

### 3.1 Clinical Characteristics of Patients

A total of 256 patients with lingual BMS were included in this study. The mean age of the study population was 64.66 ± 11.78 years. Of the 256 patients, 228 were female (89.06%) and 28 were male (10.93%), demonstrating a marked female predominance. Age distribution analysis showed that the number of female patients increased after the fifth decade of life, with the highest proportion observed in those aged 60–79 years (53.52%). Among male patients, a relatively higher proportion was observed in individuals aged ≥ 70 years (6.25%).

Regarding disease duration, the largest proportion of patients (39.8%) had symptoms for 3–12 months. The remaining patients were distributed across longer duration categories (12–24 months, 17.2%; 24–36 months, 13.7%; 36–48 months, 11.3%; 48–60 months, 4.7%; and ≥ 60 months, 13.3%).

The patients were classified according to their pain distribution patterns based on pain laterality, extent of pain and presence of central tongue involvement (Figure 1). The unilateral and bilateral pain subgroups included 46 and 128 patients, respectively. The localised and generalised pain subgroups comprised 165 and 91 patients, respectively. In addition, the non‐central involvement and central involvement subgroups consisted of 99 and 157 patients, respectively. Across all pain distribution subgroups, patients in their 60s represented the largest age group.

### 3.2 Comparison According to the Pain Laterality

Comparison of the clinical characteristics according to the pain laterality showed that the bilateral pain subgroup (*N* = 128) had a significantly higher pain intensity than the unilateral pain subgroup (*N* = 46) (VAS: 5.45 ± 2.43 vs. 4.39 ± 2.23, *p* < 0.05) (Figure 2).

**FIGURE 2:**
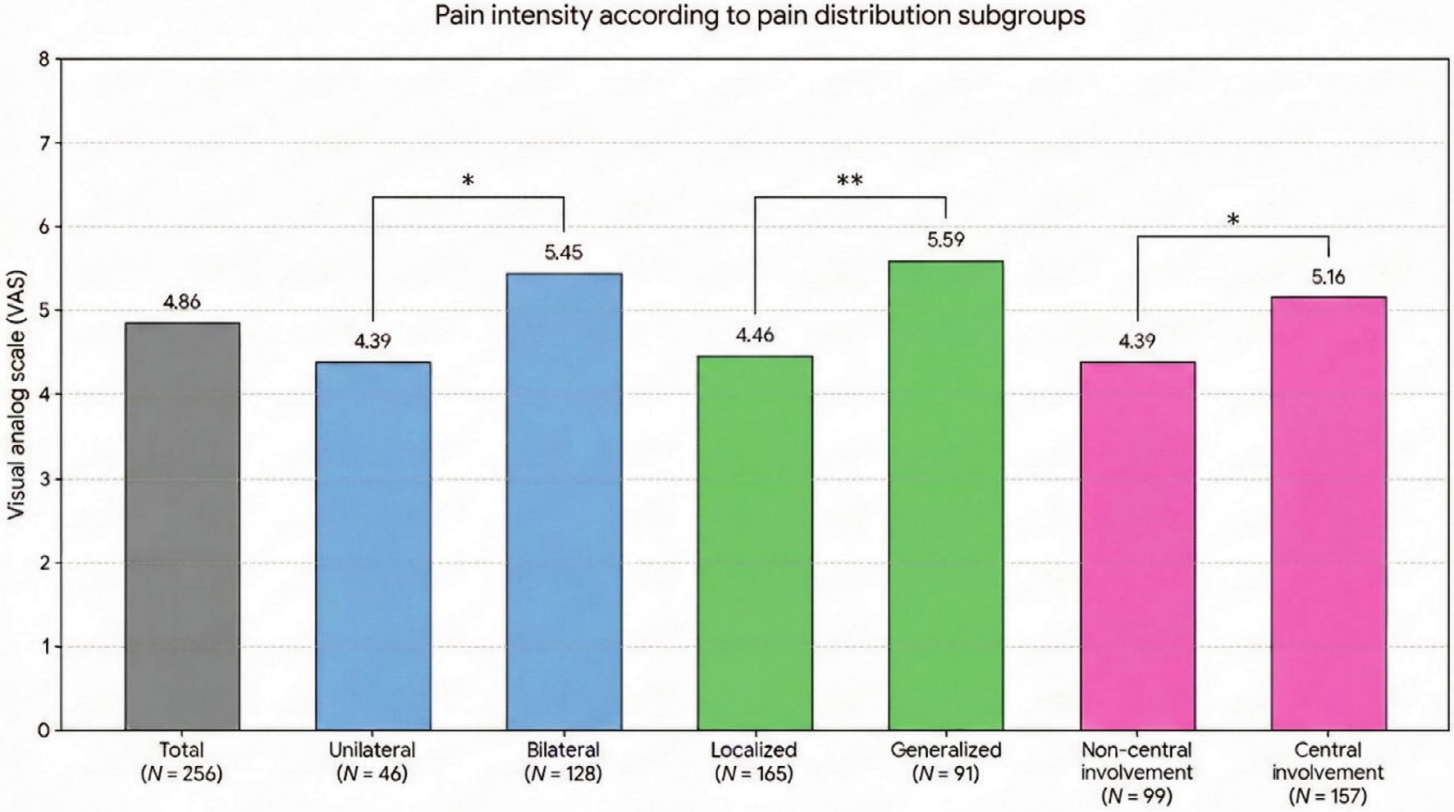
Comparison of pain intensity according to tongue pain distribution subgroup. Pain intensity was evaluated using the visual analog scale (VAS). The bars represent the mean VAS scores for the total patient cohort and each paired subgroup (unilateral vs. bilateral, localised vs. generalised, non‐central involvement vs. central involvement). An independent *t*‐test was used to compare the mean differences between the two groups within each category. *p < 0.05, **p < 0.01.

Analysis of the association between VAS and OHIP‐14 scores in the 174 patients with pain laterality (unilateral and bilateral) demonstrated a significant negative association between VAS and OHIP‐14 scores (*p* < 0.001). In the subgroup analysis, a similar significant negative association was observed in the bilateral pain subgroup (*p* < 0.05). In contrast, no significant association between VAS and OHIP‐14 scores was identified in the unilateral pain subgroup (*p* > 0.05).

Analysis of factors associated with clinically significant pain (VAS ≥ 4) showed that the presence of taste disturbance was significantly associated with clinically significant pain in the unilateral pain subgroup (*p* < 0.05). In contrast, none of the evaluated variables, including taste disturbance, globus sensation or hyposalivation, were identified as significant predictors of clinically significant pain in the bilateral pain subgroup (Table 1).

**TABLE 1:** Factors associated with clinically significant pain according to the pain laterality.

Characteristic	Unilateral tongue pain (*N* = 46)			Bilateral tongue pain (*N* = 128)		
	OR	95% CI	*p* ^a^	OR	95% CI	*p* ^a^
Taste disturbance	9.88	1.06–92.33	0.044*	1.07	0.44–2.59	0.884
Globus sensation	1.94	0.48–7.77	0.352	2.05	0.48–5.27	0.137
Hyposalivation	1.31	0.36–4.86	0.683	1.54	0.36–3.55	0.313

Abbreviations: CI, confidence interval; OR, odds ratio.

^a^ The *p*‐value was calculated by binary logistic regression analysis.

* *p* < 0.05.

### 3.3 Comparison According to the Extent of Pain

Comparison of the clinical characteristics according to the extent of pain revealed that the generalised pain subgroup (*N* = 91) showed significantly higher pain intensity than the localised pain subgroup (*N* = 165) (VAS: 5.59 ± 2.40 vs. 4.46 ± 2.30, *p* < 0.001) (Figure 2).

Analysis of the association between VAS and OHIP‐14 scores in all 256 patients demonstrated a significant negative association between VAS and OHIP‐14 scores in the overall study population (*p* < 0.05). Subgroup analyses further showed significant negative associations between VAS and OHIP‐14 scores in both the localised pain subgroup (*p* < 0.001) and the generalised pain subgroup (*p* < 0.05).

Analysis of factors associated with clinically significant pain (VAS ≥ 4) indicated that taste disturbance was significantly associated with clinically significant pain in the localised pain subgroup (*p* < 0.01). However, in the generalised pain subgroup, none of the evaluated variables, including taste disturbance, globus sensation or hyposalivation, were identified as significant predictors of clinically significant pain (Table 2).

**TABLE 2:** Factors associated with clinically significant pain according to the extent of pain.

Characteristic	Localised tongue pain (*N* = 165)			Generalised tongue pain (*N* = 91)		
	OR	95% CI	*p* ^a^	OR	95% CI	*p* ^a^
Taste disturbance	2.83	1.29–6.20	0.009**	0.57	0.20–1.62	0.288
Globus sensation	1.41	0.68–2.92	0.350	2.72	0.89–8.39	0.081
Hyposalivation	1.27	0.65–2.47	0.487	2.10	0.75–5.85	0.156

Abbreviations: CI, confidence interval; OR, odds ratio.

^a^ The *p*‐value was calculated by binary logistic regression analysis.

** *p* < 0.01.

To further investigate the relationship between pain intensity and the spatial extent of symptoms, Spearman correlation analysis was performed between VAS scores and the number of involved tongue regions. The analysis demonstrated a weak but significant positive correlation between pain intensity and the number of involved tongue regions (Spearman's *ρ* = 0.245, *p* < 0.001), suggesting that wider symptom distribution may partially contribute to greater pain intensity.

### 3.4 Comparison According to the Presence of Central Tongue Involvement

Comparison of the clinical characteristics according to the presence of central tongue involvement demonstrated that the central involvement subgroup (*N* = 157) showed significantly higher pain intensity than the non‐central involvement subgroup (*N* = 99) (VAS: 5.16 ± 2.35 vs. 4.39 ± 2.40, *p* < 0.05) (Figure 2).

Similar to the findings in the overall population, significant negative associations between VAS and OHIP‐14 scores were observed in both the non‐central involvement subgroup and the central involvement subgroup (both *p* < 0.05).

Analysis of factors associated with clinically significant pain (VAS ≥ 4) revealed that hyposalivation was significantly associated with clinically significant pain in the central involvement subgroup (*p* < 0.05). In contrast, in the non‐central involvement subgroup, none of the evaluated variables, including taste disturbance, globus sensation or hyposalivation, were identified as significant predictors of clinically significant pain (Table 3).

**TABLE 3:** Factors associated with clinically significant pain according to the presence of central tongue involvement.

Characteristic	Non‐central involvement (*N* = 99)			Central involvement (*N* = 157)		
	OR	95% CI	*p* ^a^	OR	95% CI	*p* ^a^
Taste disturbance	2.01	0.68–5.96	0.206	1.36	0.64–2.92	0.425
Globus sensation	2.17	0.83–5.62	0.112	1.56	0.73–3.35	0.256
Hyposalivation	0.84	0.36–2.00	0.700	2.08	1.01–4.27	0.047*

Abbreviations: CI, confidence interval; OR, odds ratio.

^a^ The *p*‐value was calculated by binary logistic regression analysis.

* *p* < 0.05.

## 4 Discussion

In this study, the clinical characteristics of 256 Korean patients with lingual BMS were analysed according to their pain distribution patterns, and subgroup comparisons were performed. The results demonstrated that the VAS score was significantly higher in patients with bilateral pain, generalised pain and central tongue involvement. The findings further showed that patients with a wider distribution of pain exhibited greater pain intensity, which was significantly associated with poorer oral health‐related quality of life, reflected by lower OHIP‐14 scores. Additionally, taste disturbance was significantly associated with clinically significant pain in patients with unilateral and localised pain, whereas hyposalivation was associated with clinically significant pain in patients with central tongue involvement. These findings suggest that alterations in sensory function and oral environment may contribute to the experience of pain in patients with lingual BMS. Collectively, the present results suggest that lingual BMS may exhibit clinical heterogeneity according to pain distribution patterns. Although the present findings are insufficient to establish a formal distribution‐based classification system, assessment of pain distribution may help identify clinically distinct phenotypes with different symptom characteristics and potentially different underlying mechanisms. Such phenotypic stratification may contribute to more individualised diagnostic evaluation and therapeutic approaches in patients with lingual BMS.

Herein, female patients accounted for 89.06% of the study population, with the highest proportion observed among women aged 60–69 years. This finding is consistent with previously reported findings indicating an increased prevalence of BMS in older and postmenopausal women [9].

Recent research suggests that BMS may involve both peripheral neuropathy and central nervous system dysfunction [10]. However, unlike classic neuropathic pain disorders defined by the International Association for the Study of Pain (IASP), the pain distribution in BMS often does not clearly correspond to a specific neuroanatomical territory [11, 12]. In addition, the pathophysiology of BMS remains incompletely understood and treatment responses are highly variable. Although several therapeutic approaches, including cognitive behavioural therapy, topical clonazepam, capsaicin and low‐level laser therapy, have shown potential benefits, their long‐term effectiveness and safety remain limited [13]. These findings suggest that BMS likely involves multiple interacting mechanisms, including peripheral, central and psychosocial factors, highlighting the need for more individualised clinical approaches [14].

In the present study, a comparison of clinical characteristics according to pain distribution patterns in patients with lingual BMS revealed that pain presentation frequently did not correspond to a specific neuroanatomical distribution. These findings suggest that lingual BMS may encompass pathophysiologically heterogeneous subtypes rather than represent a condition explained by a single underlying mechanism, thereby emphasising the need for refined patient stratification and individualised treatment approaches based on pain patterns and clinical characteristics.

### 4.1 Clinical Associations Between Pain Distribution and Pain Intensity

Comparisons of pain intensity according to pain distribution demonstrated that patients with bilateral pain, generalised pain and central involvement exhibited significantly higher VAS scores than patients with unilateral pain, localised pain and non‐central involvement, respectively. These findings suggest that a broader distribution of pain is associated with increased subjective pain intensity, potentially reflecting the involvement of central sensitisation in the pathophysiology of BMS, as proposed previously [15]. Neuroimaging studies have indicated that pain in BMS cannot be explained solely by peripheral nerve injury but may instead be associated with central nervous system hypersensitivity and the interaction of multiple contributing factors that amplify the pain experience [16]. Recent research implies that patients with BMS may share clinical features associated with central sensitisation, including widespread pain distribution, nonspecific systemic symptoms (e.g., fatigue, sleep disturbance and cognitive impairment) and comorbidity with other chronic pain conditions [15]. These findings may explain the observed association between pain distribution and subjective pain intensity. In addition, pain intensity showed a weak but significant positive correlation with the number of involved tongue regions. This finding suggests that the greater pain severity observed in patients with generalised symptom distribution may partly reflect spatial summation associated with wider symptom involvement, rather than exclusively indicating a distinct underlying neuropathic mechanism. Unilateral oral burning pain may clinically overlap with painful trigeminal neuropathy, particularly lingual nerve neuropathy associated with dental procedures [17]. In the present study, patients with unilateral pain more frequently reported preceding dental treatment, suggesting that some unilateral cases may share neuropathic mechanisms rather than representing classic primary BMS alone. Although patients with clinically identifiable traumatic neuropathy were excluded based on clinical history and examination findings, standardised neurosensory testing was not systematically performed because of the retrospective study design. Therefore, the possibility of subclinical trigeminal neuropathy could not be completely excluded and the findings in the unilateral pain subgroup should be interpreted with caution [18].

### 4.2 Clinical Significance of Pain Severity and Quality of Life Impairment

An increase in pain intensity was not limited to subjective discomfort but was also closely associated with deterioration in quality of life in the present study. In most subgroups, except the unilateral pain group, higher pain intensity showed a significant negative association with OHIP‐14 scores. These findings suggest that the aggravation of pain in patients with lingual BMS affects their overall quality of life, encompassing oral function impairments, such as difficulties in speech articulation and discomfort during eating and emotional and psychological burdens [19]. Similarly, prior research revealed that greater pain sensitivity was associated with poorer quality of life in patients with BMS [20]. In addition, research conducted in European populations demonstrated that oral health‐related quality of life was significantly more impaired in patients with BMS than in healthy controls and showed clinically meaningful improvement following treatment [19]. These findings emphasise the importance of assessing quality of life and pain intensity simultaneously to achieve a more comprehensive understanding of the clinical status of patients with lingual BMS.

### 4.3 Clinical Roles of Taste Disturbance and Hyposalivation According to Tongue Pain Distribution

In the present study, the presence of taste disturbance, including taste hypersensitivity and dysgeusia, was significantly associated with clinically significant pain (VAS ≥ 4) in the unilateral pain and localised pain subgroups. However, this association was not observed in the bilateral pain or generalised pain subgroups. This finding suggests that taste dysfunction is closely associated with increased pain intensity in patients presenting with localised pain patterns. A higher prevalence of taste disturbance has been reported previously in patients with bilateral pain [4], however, the present study differs in that it focused on whether the presence of taste disturbance was clinically associated with increased pain severity. Furthermore, Eliav et al. [21] demonstrated impaired chorda tympani nerve function in patients with BMS using electrogustometry and stated that even unilateral dysfunction may induce burning pain across the tongue. Additional evidence suggests that reduced lingual nerve input may contribute to both taste disturbance and pain pathophysiology in BMS [22]. These previous findings support the association between taste disturbance and increased pain intensity that was observed in patients with localised pain in the current study. However, as the taste disturbance assessment in this study was based on subjective patient reports and did not include quantitative gustatory testing, the results should be interpreted with caution.

Analysis of salivary parameters demonstrated that hyposalivation was associated with clinically significant pain in the central involvement subgroup. In the present study, the USFR was evaluated using standardised measurement procedures recommended by the American Dental Association diagnostic guidelines [23]. It has previously been reported that xerostomia may increase the subjective pain sensitivity and contribute to an impaired quality of life in patients with BMS [24]. Although many patients with BMS report symptoms of xerostomia, an objective reduction in salivary flow has not been consistently observed [25]. This study provides preliminary evidence that objectively measured changes in salivary flow may be differentially associated with pain severity according to the pain distribution pattern, thereby contributing to a better understanding of the clinical heterogeneity of lingual BMS. The relationship between pain distribution patterns and disease duration may also be relevant to the progression of BMS. More widespread or bilateral pain patterns may reflect progressive central sensitisation during the chronic course of the disease [12, 15], whereas taste disturbance may be more closely associated with localised or earlier‐stage sensory dysfunction.

### 4.4 Limitations

This study has some limitations. First, because this was a retrospective cross‐sectional study conducted at a single centre, the possibility of selection bias cannot be excluded and the generalisability of the findings to broader populations or other clinical settings may be limited. Additionally, causal relationships among pain intensity, quality of life and oral environmental parameters could not be directly established. Second, the relatively small sample sizes in certain subgroups, including the unilateral pain and non‐central involvement groups, may have introduced statistical uncertainty, warranting cautious interpretation and generalisation of the findings. Another limitation of the present study is that it focused specifically on tongue pain distribution patterns and did not systematically evaluate pain involving other intraoral sites such as the gingiva, palate or lips. Therefore, the terms ‘localised’ and ‘generalised’ should be interpreted within the context of tongue pain distribution rather than the entire oral cavity. Furthermore, more detailed anatomical subclassifications, including tip‐only or anterior tongue involvement, were not separately analysed because further subgroup stratification may have reduced statistical robustness. Additionally, potential confounding variables, such as age, sex and disease duration, were not adjusted for in the regression analyses, which may have influenced the observed associations. Moreover, menopausal status was not consistently available in the retrospective medical records and therefore could not be included in the analyses, despite its potential relevance to BMS. Finally, standardised neurosensory examinations for trigeminal neuropathy were not systematically performed because of the retrospective nature of the study. Although patients with clinically identifiable traumatic neuropathy were excluded during clinical evaluation, the possibility of subclinical neuropathic conditions, particularly in unilateral pain cases with preceding dental treatment, cannot be completely excluded.

To address these limitations, future prospective studies with larger sample sizes are required to validate the identified clinical differences according to pain distribution patterns. In addition, longitudinal investigations tracking temporal changes in pain intensity, quality of life, oral environmental factors and psychological variables are needed to clarify the causal relationships underlying the observed associations. Such approaches may further improve our understanding of the pathophysiology of lingual BMS and strengthen the evidence base for pain distribution‐based clinical assessments and individualised management strategies.

## 5 Conclusion

To our knowledge, this study is the first large‐scale investigation to comprehensively analyse clinical and physiological characteristics according to pain distribution patterns in a cohort of 256 Korean patients with lingual BMS. Collectively, our findings demonstrate that pain distribution may reflect differences in key clinical indicators, including VAS score, OHIP‐14 score, taste disturbance and hyposalivation.

The present findings suggest that lingual BMS may exhibit clinical heterogeneity according to pain distribution patterns, with differences in pain intensity and associated clinical features across subgroups. Systematic evaluation of intraoral clinical factors may assist clinicians in characterising individual pain patterns and associated features, thereby facilitating more appropriate and individualised treatment and management strategies for patients with lingual BMS.

## Author Contributions

**Jae‐Woo Park:** methodology, writing – review and editing. **Na‐Yeon Ha:** investigation, data curation, formal analysis, writing – original draft. **Yoohyun Sim:** conceptualization, visualization, investigation, data curation, formal analysis, writing – original draft. **Jinsung Kim:** methodology, writing – review and editing, supervision. **Seok‐Jae Ko:** methodology, writing – review and editing.

## Funding

This work was supported by Korea Health Industry Development Institute (Grant RS‐2024‐00443769).

## Conflicts of Interest

The authors declare no conflicts of interest.

## Data Availability

The data that support the findings of this study are available from the corresponding author upon reasonable request.
